# The Functional DNA Methylation Signatures Relevant to Altered Immune Response of Neonatal T Cells with l-Arginine Supplementation

**DOI:** 10.3390/nu13082780

**Published:** 2021-08-13

**Authors:** Hong-Ren Yu, Te-Yao Hsu, Ching-Chang Tsai, Hsin-Chun Huang, Hsin-Hsin Cheng, Yun-Ju Lai, Yu-Ju Lin, Chih-Cheng Chen, Sung-Chou Li, Kuender Yang

**Affiliations:** 1 Department of Pediatrics, Kaohsiung Chang Gung Memorial Hospital and Chang Gung University College of Medicine, Kaohsiung 833401, Taiwan; yuu2004taiwan@yahoo.com.tw (H.-R.Y.); hhuang@cgmh.org.tw (H.-C.H.); charllysc@adm.cgmh.org.tw (C.-C.C.); 2 Department of Obstetrics and Gynecology, Kaohsiung Chang Gung Memorial Hospital and Chang Gung University College of Medicine, Kaohsiung 833401, Taiwan; tyhsu@adm.cgmh.org.tw (T.-Y.H.); aniki@cgmh.org.tw (C.-C.T.); chokovarous@cgmh.org.tw (H.-H.C.); lusionbear@hotmail.com (Y.-J.L.); lyu015@cgmh.org.tw (Y.-J.L.); 3 Department of Medical Research, Kaohsiung Chang Gung Memorial Hospital and Chang Gung University College of Medicine, Kaohsiung 833401, Taiwan; 4 Department of Pediatrics, Mackay Memorial Hospital, Taipei 104217, Taiwan

**Keywords:** neonate, cytokines, T cells, epigenetics, l-arginine

## Abstract

l-Arginine is an important nutrient in the infant diet that significantly regulates the maturation of the immune system in neonates, including the maturation of CD4^+^ T cells. The biological activities of CD4^+^ T cells differ substantially between neonates and adults, and these differences may be governed by epigenetic processes. Investigating these differences and the causative processes may help understand neonatal and developmental immunity. In this study, we compared the functional DNA methylation profiles in CD4^+^ T cells of neonates and adults, focusing on the role of l-arginine supplementation. Umbilical cord blood and adult CD4^+^ T cells were cultured with/without l-arginine treatment. By comparing DNA methylation in samples without l-arginine treatment, we found that CD4^+^ T cells of neonatal cord blood generally showed higher DNA methylation than those of adults (average CpG methylation percentage 0.6305 for neonate and 0.6254 for adult, *t*-test *p*-value < 0.0001), suggesting gene silencing in neonates. By examining DNA methylation patterns of CpG dinucleotides induced by l-arginine treatment, we found that more CpG dinucleotides were hypomethylated and more genes appeared to be activated in neonatal T-cells as compared with adult. Genes activated by l-arginine stimulation of cord blood samples were more enriched regarding immune-related pathways. CpG dinucleotides at IL-13 promoter regions were hypomethylated after l-arginine stimulation. Hypomethylated CpG dinucleotides corresponded to higher IL-13 gene expression and cytokine production. Thus, DNA methylation partially accounts for the mechanism underlying differential immune function in neonates. Modulatory effects of l-arginine on DNA methylation are gene-specific. Nutritional intervention is a potential strategy to modulate immune function of neonates.

## 1. Introduction

Discrepancies in biomolecules of immune cells between newborns and adults contribute to differences in microbial susceptibility and atopic properties [1,2,3,4,5]. Such immune differences are manifested in differential cell subset constitutions, immune responses, and distinct cellular or humoral molecules. In a previous study, some proteins differentially abundant between adult and neonatal mononuclear cells were identified. Among them, adenosine deaminase decreased and arginase-1 increased in neonatal mononuclear cells, which indicated the correction of impaired immune response in newborns [5]. Arginine, a semi-essential amino acid, plays a crucial role in wound healing, tissue integrity maintaining, immune function, reproduction, and fetal development [6,7,8,9,10]. Our previous study showed that neonatal blood has lower arginine concentrations than adult [11]. In premature neonates, hypoargininemia is more frequently observed [12] and hypothesized to predispose such infants to the development of necrotizing enterocolitis and respiratory distress syndrome in preterm infants [13,14]. Tomlinson et al. found that an infant receives about 45 mg/kg/d of arginine from human milk. However, assuming 2 g/kg/d net protein accretion, healthy neonates require at least 140 mg/kg/d of arginine for protein synthesis alone [15]. Arginine is supplied in quantities that are much less than the needs of the neonate in human breast milk and most infant formulas. Under stress and other catabolic conditions when the capacity of endogenous arginine synthesis is exceeded, arginine will be insufficiency. Different modulation effects of l-arginine on neonatal leukocytes have also been shown to mediate through an interleukin-2 independent pathway [11]. We further postulate that this metabolic regulation of T cell functions might be relevant to epigenetic regulations.

Epigenetics comprise numerous biological processes that alter gene expression without changing the DNA sequence. Adequate regulation of activating or silencing key genes is crucial for effective immune functions and to prevent dysfunctions such as allergies or autoimmunity [16], thus epigenetic mechanisms are most likely crucial for immune system homeostasis. In previous studies, we observed that prenatal insult can exert considerable effects on innate and adaptive immunity of the offspring through histone modification [17,18]. Recently, the essential regulatory roles of specific miRNAs in neonatal immune responses have been reviewed [19]. For example, miR-184 was reported to regulate nuclear factor of activated T cells-1 (NFAT1) in neonatal CD4 T cells [20]; miR-146a and miR-155 down-regulate Toll-like receptor signals of neonatal monocytes and plasmacytoid dendritic cells, respectively [21,22]; and miR-125b, miR-142-3p, and let-7g downregulate LPS-induced cytokine production in neonatal monocytes/polymorphonuclear leukocytes [23,24]. Functional miRNA signatures of specific immune cell subsets were also shown to differ between neonates and adults [25]. These findings may be of use to further our understanding of the differential immune response in neonates.

DNA methylation also plays an important and dynamic role in regulating gene expression. Regarding immune functions, changes in DNA methylation have been demonstrated to affect differential secretion of Th1 and Th2 cytokines [26]. Moreover, demethylation of cytosine-guanine (CpG) motifs of the forkhead box transcription factor (FOXP3) is positively correlated with its mRNA expression in CD4^+^CD25hi T cells and suppressive capacity of regulatory T cells (Treg) [27]. These insights highlight the importance of DNA methylation for T cell polarization. Few studies focused on DNA methylation variation contributing to altered immune function in neonates [28,29]. White et al. reported that neonatal CD4/CD45RO- T cells, but not CD8^+^ T cells, are hypermethylated at CpG within and adjacent to the IFN-γ promoter [28,30]. This methylation difference corresponded to impaired IFN-γ production in neonatal CD4^+^ T cells [30]; however, neonatal CD8^+^ T cells and NK cells were less methylated and were comparable with those of adults. In a previous study, we also demonstrated that l-arginine enhances IL-10 production of neonatal Treg through decreasing methylation of IL-10 promoter [31]. Interestingly, the function promoting effect of l-arginine on neonatal Treg is selective and occurs in IL-10 but not in TGF-β [31]. Immune functions differ considerably between adults and neonates due to immaturity of the neonate immune system and minimal exposure to pathogens [19]. T cells play a critical role in adaptive immunity and determining the specificity of DNA methylation profiles in T cells is important for comprehensive understanding of the neonatal immune systems. In this study, we explored the differences of functional DNA methylation profiles between PHA-stimulated neonatal and adult CD4^+^ T cells. Furthermore, we observed that certain cytokines were modulated by l-arginine through DNA methylation modification.

## 2. Materials and Methods

### 2.1. Subject Enrollment

Adult peripheral blood (AD) samples were collected from healthy volunteers aged 20–40 years, and umbilical cord blood (CB) samples were collected with informed consent from cases of elective cesarean section or normal spontaneous delivery by healthy mothers (Table 1). Pregnant women with pregnancy related complication such as gestational diabetes, hypertension, preeclampsia, preterm labor or gestational infection were excluded. In summary, we collected 15 neonatal cord blood samples and 15 adult peripheral blood samples for CD4^+^ T cell purification. For each sample, half of the collected CD4^+^ cells were subjected to l-arginine treatment and the remaining half were not. As a result, we collected 15 sets of cord blood CD4^+^ cells without l-arginine treatment (CC set), 15 sets of cord blood CD4^+^ cells with l-arginine treatment (CT set), 15 sets of adult CD4^+^ cells without l-arginine treatment (AC set) and 15 sets of adult CD4^+^ cells with l-arginine treatment (AT set). Among the collected CD4^+^ cells, a fraction of them (three CC, three CT, three AC and three AT sets) was used for DNA extraction and M850 assays to determine DNA methylation. The remaining sets of CD4^+^ cells (12 CC, 12 CT, 12 AC and 12 AT sets) were used for qPCR and ELISA assays. This study was approved by the institutional ethics board (IRB number: 103-3725A3 and 20170204A3) of Kaohsiung Chang Gung Memorial Hospital and was carried out in accordance with the regulations.

We first identified significantly methylated CpG dinucleotides in comparisons. Then, by mapping the CpG dinucleotides back to genome, we identified the genes whose promoter regions were located by the mapped CpG dinucleotides. Finally, pathway enrichment analysis identified the pathways significantly enriched by the genes according to KEGG annotation. The detailed pathway information was available in Supplementary Tables S2–S4. The *p*-value of pathway enrichment analysis is calculated based on hypergeometric distribution.

### 2.2. Cell Enrichment, Cell Culture and l-Arginine Treatment

Whole blood samples were obtained from peripheral blood vessels or from the umbilical cord, followed by CD4^+^ T cells enrichment by using BD IMag™ anti-human CD4 Particles (Cat # 557767, BD, Franklin Lakes, NJ, USA) as described previously [3,11,25]. In summary, in each run of enrichment, 10^7^ leukocyte cells and 50 μL CD4^+^ beads were mixed thoroughly in a tube and incubated at room temperature for 30 min. Then, 1 mL BD IMag buffer was added and the solution was transferred into FACS tube. By placing the FACS tube in strong magnetic field for 8–10 min, CD4^+^ T cells attached onto the internal wall of the tube so that the supernatants could be carefully removed. Next, the tube was placed out of the magnetic field and washed twice with the IMag buffer to collect CD4^+^ T cells. The purity of CD4^+^ T cells was further confirmed to be >95% by flow cytometry. CB or adult CD4^+^ T cells (2 × 10^6^ cells per mL) were stimulated with purified phytohemagglutinin (PHA, 10 µg/mL; L1668, Sigma-Aldrich, St. Louis, MO, USA) in 1-cm culture plates with l-arginine-free medium (SILAC R1780 SIGMA RPMI-1640 Medium; Sigma-Aldrich) and 10% heat-inactivated fetal bovine serum, 1 mM glutamine, 1 mM sodium pyruvate, 50 mg/L, l-leucine, 40 mg/L, l-lysine, and 1 × non-essential amino acids (Gibco; Thermo Fisher Scientific, Waltham, MA, USA), 100 IE/mL penicillin. For the differential treatment, CB or adult CD4^+^ T cells were stimulated in the medium containing phytohemagglutinin (PHA) with exogenous supplementation of 1150 μM l-arginine (Sigma-Aldrich), the l-arginine concentration in the commercialized regular RPMI (https://www.thermofisher.com/tw/zt/home/technical-resources/media-formulation.114.html, accessed on 1 July 2021). In total, four experimental sets were used: CB CD4^+^ T cells without l-arginine treatment (set CC), CB CD4^+^ T cells with l-arginine treatment (set CT), adult CD4^+^ T cells without l-arginine treatment (set AC), and adult CD4^+^ T cells with l-arginine treatment (set AT). After 48 h, cell pellets and supernatants were collected for methylation and mRNA assays and cytokine production assays. The cell culture supernatants were used for measuring IFN-γ, IL-4, IL-17A and IL-13 using ELISAs.

### 2.3. DNA Methylation Assay

CD4^+^ cells of the four experimental sets (CC, CT AC and AT) were first subjected to DNA extraction with the QIAamp^®^ DNA Blood Mini Kit (Qiagen, Hilden, Düsseldorf, Germany) to extract DNA by following the manufacturer’s instructions. Then, the collected DNA samples were further subjected to bisulfite conversion with EZ DNA Methylation-Lightning ^TM^ Kit (Zymo Research, Irvine, CA, USA). Briefly, 0.5 g of DNA per reaction was mixed with lightning conversion reagent for reactions, followed by being loaded into spin column and mixed with M-binding buffer for centrifuge. The Bisulfite-converted DNA samples were used for genome-wide examination of DNA methylation using the Infinium MethylationEPIC BeadChip (M850K assay; Illumina, San Diego, CA, USA) which measures methylation proportions (termed β-values) of approximately 850,000 CpG dinucleotides. Three independent samples of each treatment set were used in the M850K assay, resulting in methylation data of 12 samples. Significantly methylated CpG dinucleotides were conducted with ANOVA by using Partek (Qiagen, Hilden, Germany) to test the differences in β-values between sets CC and AC. DNA methylation data was analyzed with Partek (Qiagen, Germany) to identify the significantly methylated CpG dinucleotides.

### 2.4. Reverse-Transcription qPCR and ELISAs

To examine gene expression variation, we conducted qPCR assays. In summary, total cellular RNA was extracted using TRIzol Reagent (Invitrogen, Carlsbad, CA, USA) according to the manufacturer’s instruction. A total of 100 ng of collected RNA was subjected to reverse transcription for 60 min at 42 °C. Then, the cDNA products were subjected to PCR amplification with specific primers and SYBR GREEN master mix quantification for amplifying. The Sequences of the PCR primers are shown in Supplementary Table S1. The PCR steps were activated by heating for 10 min at 95 °C for 1 cycle, then for 15 s at 95 °C, and finally for 60 s at 60 °C for 40 cycles in a PCR mix containing 2 µL of the cDNA template, 0.5 µL of 10 µM primer, and 5 µL of SYBR GREEN master mix in a total volume of 20 µL. These PCR assays included a negative control without a template to ensure absence of contamination. All reactions were performed in triplicate in an ABI 7500 sequence detection system (Applied Biosystems, Perkin-Elmer, Foster City, CA, USA). In addition to mRNA levels of the interested genes, we also applied ELISA to measure the concentrations of the interested proteins secreted by CD4^+^ T cells. In summary, 72 h after culture, cell-free culture supernatants were collected and assayed for cytokine production with Enzyme-linked immunosorbent assay (ELISA). In this study, four proteins were measured with individual ELISA kit as follow: IL-4 (Cat # BMS225HS, eBioscience, Vienna, Austria), IL-17A (Cat # 88-7176-88, eBioscience), IFN-γ (Cat # 88-7316-88, eBioscience) and IL13 (Cat #DY213, R&D Systems Inc., Minneapolis, MN, USA). We conducted ELISAs by following the manufacturers’ instructions.

### 2.5. Statistics Analysis

The Mann–Whitney U test or Fisher’s *t*-test was used to calculate the *p*-values. Results with a *p*-value of less than 0.05 were considered to be statistically significant. All statistical tests were performed using the SPSS 22.0 for Windows XP (SPSS, Inc., Chicago, IL, USA). Data was presented as mean ± SEM.

## 3. Results

### 3.1. Correlation of Overall DNA Methylation Patterns with CC and AC Treatments

**Figure 1:**
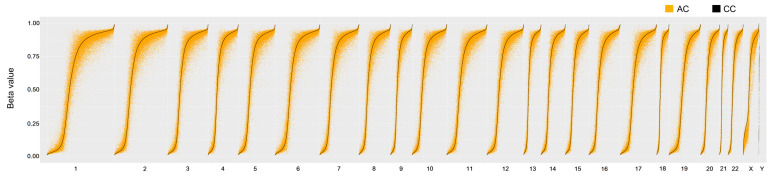
Scatter plot of neonatal and adult CpG methylation in chromosomes. We determined β-values (methylation proportion) of CpG dinucleotides using M850K assays. Each CpG dinucleotide has one βcc and one βac value. The former and the latter were derived from three CC and three AC samples, respectively, without l-arginine treatment. For each chromosome, we tabulated βcc and one βac values by sorting the βcc values. Then, we made scatter plots (*X*-axis: βcc; *Y*-axis: βac). Although overall highly correlated, βcc and βac values of specific CpG dinucleotides can vary substantially. CC and CT refer to cord blood CD4^+^ T cells without and with l-arginine treatment, respectively. AC and AT refer to adult blood CD4^+^ T cells without and with l-arginine treatment, respectively.

We enrolled subjects for collecting adult peripheral-blood CD4+ T cells or neonatal cord-blood CD4^+^ T cells. We examined differences in DNA methylation patterns by using DNA methylation microarray assays between CC and AC sets and, focusing on the intrinsic variation due to l-arginine stimulation in adult and neonate samples. As a result, each CpG dinucleotide had two average β-values (the methylation percentage of a CpG dinucleotide, βcc from the three CC and βac from the three AC samples). Then, we categorized them based on the βcc value. We produced X-Y scatter plots for each chromosome. In total, 82,014 CpG dinucleotides occurred in chromosome 1, and the data were plotted based on the sorted βcc values (Figure 1; *x*-axis: βcc values; *y*-axis: βac values). Figure 1 shows that the patterns of 22+XY chromosomes were similar, and overall βcc and βac (*y*-axis) were highly correlated in chromosomes. For a more comprehensive comparison, we re-sorted the β-values without considering chromosome difference and produced a plot (Supplementary Figure S1). A similar pattern as in Figure 1 was observed, and a strong positive correlation between βcc and βac was confirmed (Pearson’s *r* = 0.9874, *p* < 0.0001).

### 3.2. Methylation Proportions of CpG Dinucleotides in CC and AC

**Figure 2:**
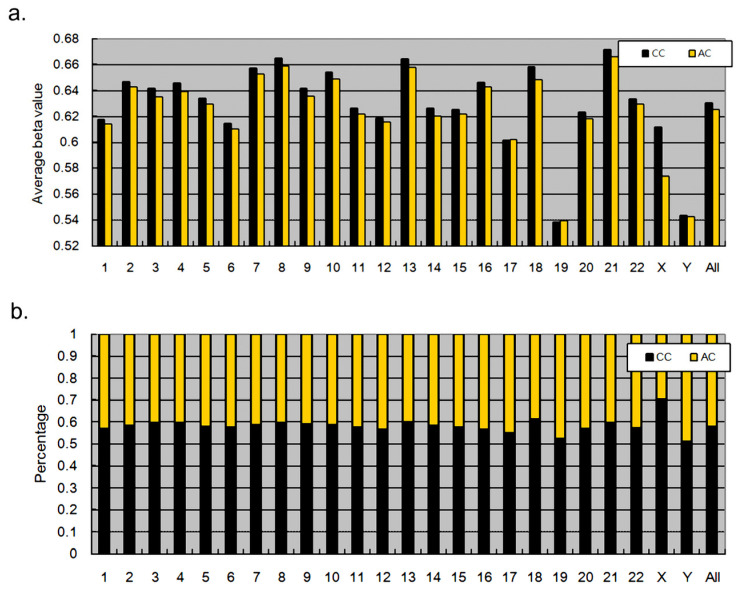
Comparisons of neonatal and adult CpG methylation in chromosomes. (**a**) The comparison was conducted based on the average β-values of CpG dinucleotides in each chromosome. (**b**) The comparison was conducted based on the percentage of cases in which βcc was larger than βac. In the ‘All’ set, this βcc > βac tendency was significant (Fisher’s *t*-test, *p* < 0.001).

Despite the strong correlation of βcc and βac, Figure 1 shows several data points (CpG dinucleotides) with substantial variation between βcc and βac. We thus examined the intrinsic differences in DNA methylation between CC and AC treatment sets. For CpG dinucleotides in each chromosome, we first calculated the average β-values of each chromosome. For examples, the 82,014 CpG dinucleotides in chromosome 1 resulted in an average β-value of 0.6175 in CC and in an average β-value of 0.6140 in AC (Figure 2a). By comparing 24 chromosomes, we found that the CpG dinucleotides in set CC generally produced higher average β-values than in set AC in most chromosomes (except for chromosomes 17 and 19), suggesting higher methylation levels in CpG dinucleotides of neonatal T cells compared to those of adults. We further conducted a more detailed comparison by calculating the proportion of cases in which βcc was larger than βac for all CpG dinucleotides in each chromosome. For example, 56.85% of the 82,014 CpG dinucleotides in chromosome 1 had higher β-values in set CC than in set AC (Figure 2b). This trend was consistently observed in all chromosomes, including chromosome 17 and 19, and the CC > AC difference was significant (*p* < 0.0001).

### 3.3. Genes with Significantly Different CpG Dinucleotides between CC and AC Samples Were Mainly Involved in Cell Adhesion and Metabolism

**Figure 3:**
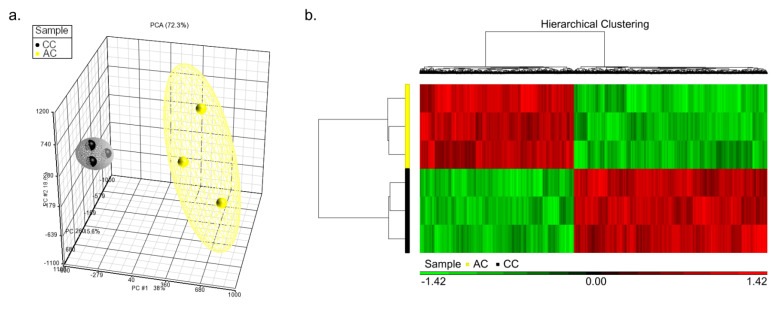
ANOVA of neonatal and adult CpG methylation. We compared the three CC and three AC M850K microarray data using Partek. (**a**) The PCA plot demonstrated substantial discrimination between CC and AC samples. (**b**) At FDR < 0.05 and β variation > 0.1, 33,150 CpG dinucleotides differed significantly, 44.34% of which were hypermethylated in treatment AC.

Apart from general differences in DNA methylation, we also identified genes with significant differences in CpG dinucleotides between sets CC and AC. As shown in a principal component analysis plot (Figure 3a) based on DNA methylation profiles, the CC samples were clearly separated from the AC samples, suggesting high discrimination between CC and AC samples. Specifying false discovery rate (FDR) < 0.05 and β-variation > 0.1, 33, 150 CpG dinucleotides showed significant variation with 14,699 CpG dinucleotides hypermethylated and 18,451 CpG dinucleotides hypomethylated in AC samples (Figure 3b).

To identify the genes regulated by these significant CpG dinucleotides, CpG dinucleotides were mapped back to the genome, and we examined whether they were located at the putative promoter regions (−5000 upstream to +3000 downstream of transcription start site) of genes as suggested in a previous study [32]. As a result, 11,660 CpG dinucleotides were found to be located at the respective promoter regions of 7730 genes. We further conducted a pathway enrichment analysis on these genes using Partek. As shown in Supplementary Table S2, 24 significant pathways were identified, and most of them were associated with synthesis of cell adhesion molecules and metabolic functions. The summary of significantly altered CpG dinucleotides, genes and enriched pathways was also available in Table 1.

### 3.4. Expression Intensity and DNA Methylation of T Cell-Specific Genes

**Figure 4:**
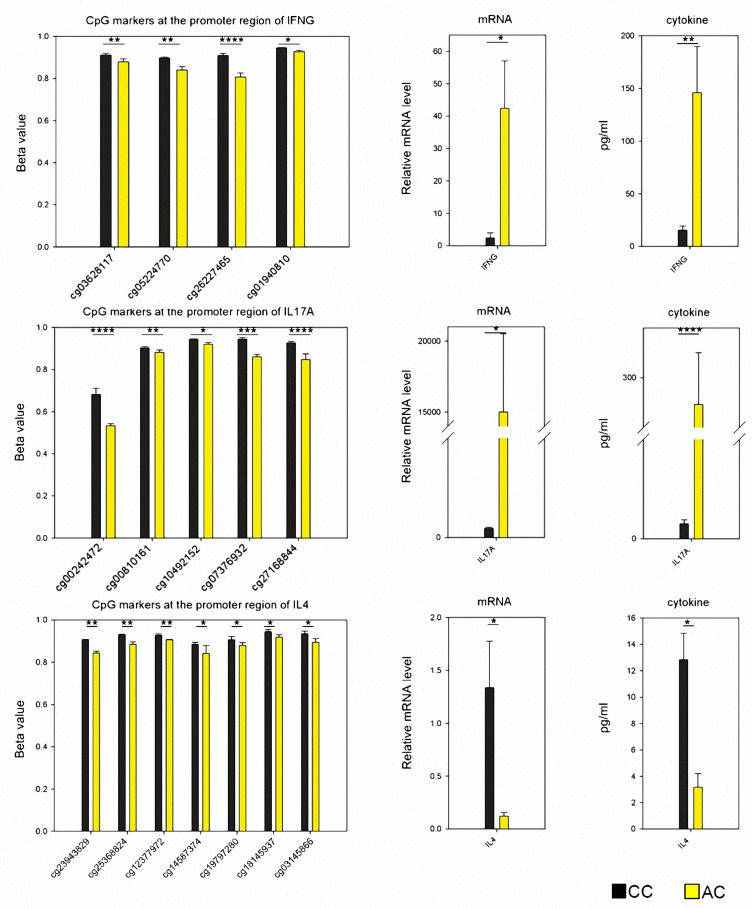
CpG methylation profile, mRNA expression, and protein production of CD4^+^-specific genes. Among genes with examined CpG dinucleotides, IFNG, IL-17A, and IL-4 were CD4^+^ T cell-specific; we thus compared CpG methylation profiles (*n* = 3, *t*-test), mRNA expression (qPCR data presented with 2^−ΔΔCt^ format, *n* = 12, Mann–Whitney U test), and protein production between CC and AC samples (*n* = 12, Mann–Whitney U test). *, **, ***, **** denoted *p*-value < 0.05, 0.01, 0.001 and 0.0001, respectively.

Since hypermethylation in DNA typically leads to lower gene expression, we tested whether such relationships occurred in CD4^+^-specific genes associated with T cells polarization, including IFNG, IL-17A, and IL-4. Between CC and AC samples, we first tabulated the significantly different CpG dinucleotides located within the putative promoter regions (−5000 upstream to +3000 downstream of the transcription start site). Then, we compared gene expression in CD4^+^ cells using a qPCR assay and we also measured protein concentrations in the medium using an ELISA. As shown in Figure 4, the IFNG results showed high DNA methylation in both AC and CC samples, and CC samples had a higher CpG methylation than AC samples associated with a significantly lower IFNG mRNA expression level than AC samples; in addition, the levels of protein secreted into the medium was comparative to the mRNA expression.

IL-17A which also showed higher methylated CpG dinucleotides in CC samples had lower levels of mRNA expression, and lower protein concentrations in CC samples. However, the higher IL-4 methylation levels in CC samples were associated with higher mRNA expression levels in cells and higher protein concentrations in the medium, indicating a contradictory correlation. These results implied that in addition to DNA methylation, gene expression of IL-4 was regulated also by other mechanisms.

### 3.5. Enrichment of Genes Activated by l-Arginine Treatment

Supplementary Table S2 shows that the genes with differential CpG dinucleotides between sets CC and AC (without l-arginine supplementation) were mostly associated with metabolic functions. In previous studies, we found that l-arginine can modulate neonatal lymphocyte proliferation and regulatory T cell DNA methylation [11,31]. Most genes in neonatal CD4^+^ cells seemed to be less activated than those in adult CD4^+^ cells, thus we assessed which genes and which pathways were activated by l-arginine treatment. To answer this question, we identified the significant (*p* < 0.05, *t*-test; β-difference > 0.05) CpG dinucleotides (hypomethylated with l-arginine treatment) by comparing set CT with set CC and AT with AC, after which we identified the genes regulated by these significant CpG dinucleotides. In total, 3797 and 617 CpG dinucleotides were hypomethylated in the CT vs. CC and in the AT vs. AC comparison, respectively. In addition, the former and the latter methylation patterns showed activation of 1380 and 296 genes, respectively. We conducted a pathway enrichment analysis of the genes activated by hypomethylated CpG dinucleotides after the l-arginine treatment in CT and AT samples. The complete pathway analysis results are shown in Supplementary Table S3 (CT vs. CC) and Supplementary Table S4 (AT vs. AC).

**Figure 5:**
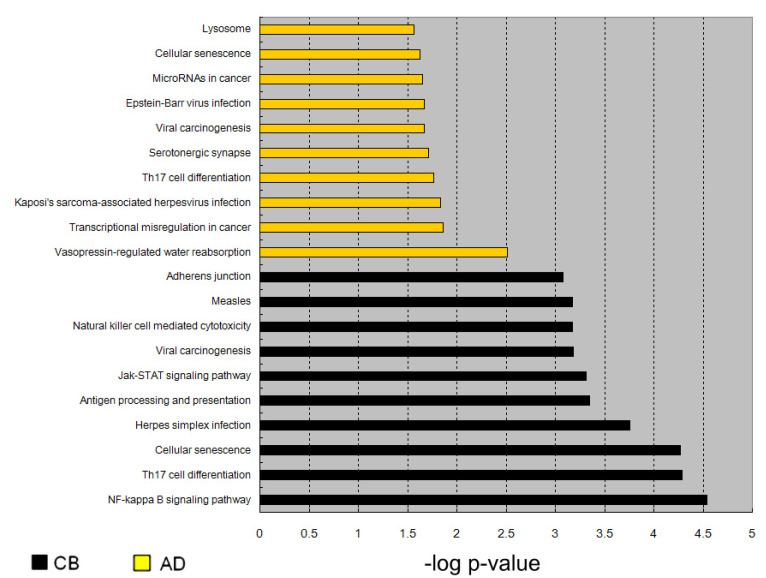
Comparisons of enriched pathways between cord blood (CB) and adult blood (AD). We identified significantly hypomethylated CpG dinucleotides (CT vs. CC, comparing the factor l-arginine treatment) and identified activated genes of cord blood and adult blood samples, followed by pathway analysis. We then compared the top-10 most significant pathways identified in either CB or AD samples.

After the l-arginine treatment, more genes were activated in set CT than in set AT, thus the pathways enriched by the CT-activated genes were substantially more significant than those enriched by the AT-activated genes. As shown in Supplementary Tables S3 and S4, 50 and 18 pathways were activated in neonatal and adults CD4^+^ T cells treated with l-arginine, respectively. Figure 5 shows the top-10 significantly activated pathways in adult and cord blood CD4^+^ T cells treated with l-arginine. Among the top-10 pathways in the CT vs. CC comparison, eight were immune-related pathways. In the AT vs. AC comparison, only three were immune-related pathways. The immune-related pathways of the CT vs. CC comparison were substantially higher significant than those of the AT vs. AC comparison. Cellular senescence, viral carcinogenesis, and Th17 cell differentiation pathways were up-regulated in both cord blood T cells and adult T cells with l-arginine treatment.

Gene ontology (GO) is typically applied to interpret potential functions of genes; we therefore also conducted GO analysis on the activated genes in CT and AT samples. In line with the pathway results, the identified GO items in CT samples were more frequently associated with immunity (Supplementary Figure S2), whereas the identified GO items in AT samples were more frequently associated with metabolism. Compared with adults, neonates are less exposed to exogenous stimulants and pathogens, thus neonatal immune functions are typically less activated than those of adults [33,34]. l-Arginine can activate T cell immune responses and is required for defense against various pathogens [35]. Neonatal T cells are more susceptible to l-arginine supplementation [31], which explains why more immune-related genes and pathways were activated in neonatal CD4^+^ cells than in those of adults. The complete GO analysis results are available in Supplementary Table S5 (CT vs. CC) and Supplementary Table S6 (AT vs. AC).

### 3.6. IL-13 Was Activated by DNA Hypomethylation in Neonatal CD4^+^ Cells Due to the l-Arginine Treatment

**Figure 6:**
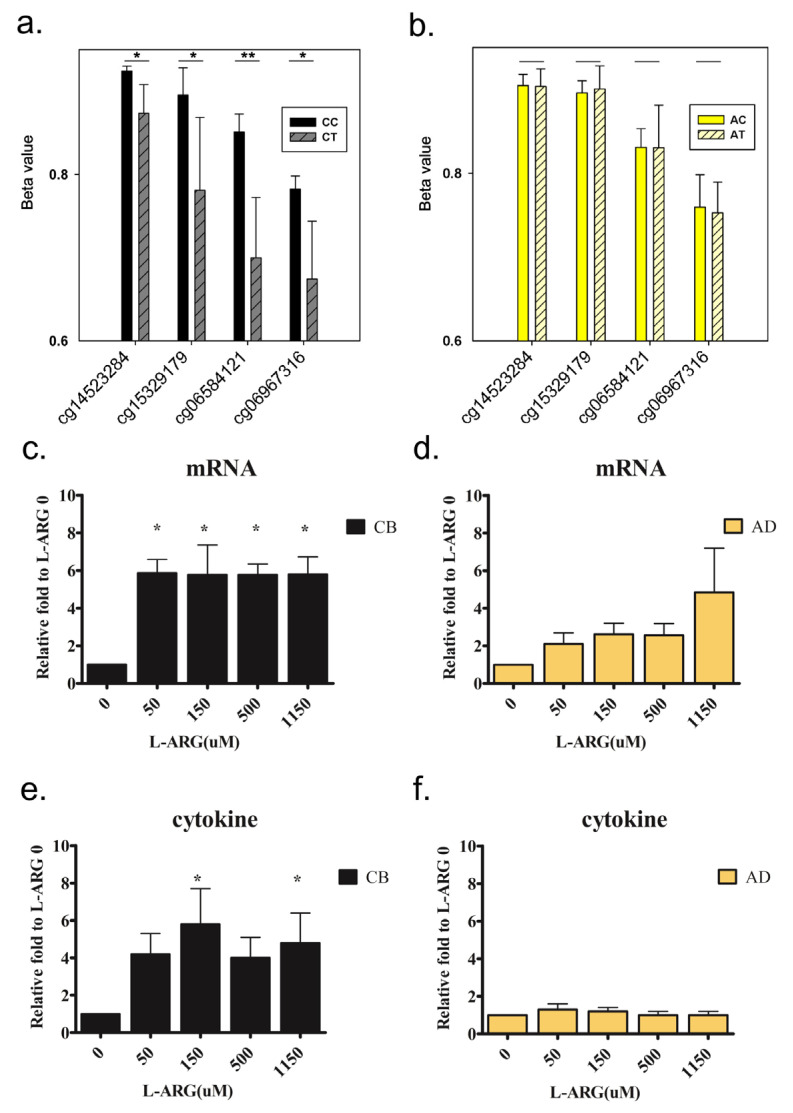
Variations of CpG methylation profiles, mRNA expression, and protein production of IL-13 in cord blood and adult blood after l-arginine treatment. We tested whether the l-arginine treatment affected the CpG methylation profile (**a**,**b**, *n* = 3, *t*-test), mRNA expression (**c**,**d**, *n* = 12, Mann–Whitney U test), and protein production (**e**,**f**, *n* = 12, Mann–Whitney U test) of IL-13 in cord blood and adult blood. * and ** denoted *p*-value < 0.05 and 0.01, respectively.

Among the genes activated by DNA hypomethylation due to the l-arginine treatment, we were particularly interested in IL-13 which has become an important therapeutic target for treating Th2-mediated diseases. Therefore, we examined to what extent IL-13 was activated by the l-arginine treatment and DNA hypomethylation. As shown in Figure 6a, four CpG dinucleotides located within the putative promoter region were significantly hypomethylated in cord blood CD4^+^ cells (CT vs. CC) due to the l-arginine treatment; however, these four CpG dinucleotides showed no significant variation after l-arginine treatment in adult CD4^+^ cells (AT vs. AC; Figure 6b).

The qPCR assays demonstrated that IL-13 mRNA was significantly increased in cord blood CD4^+^ cells after treatment with l-arginine at different concentrations (Figure 6c). This variation was not significant in adult CD4^+^ cells (Figure 6d). The protein product of the IL-13 gene is typically secreted from cells, thus we also measured IL-13 protein levels in the medium, and we observed the patterns similar to those of the mRNA measurements (Figure 6e,f), suggesting up-regulation of IL-13 by DNA hypomethylation due to treatment with l-arginine.

## 4. Discussion

**Figure 7:**
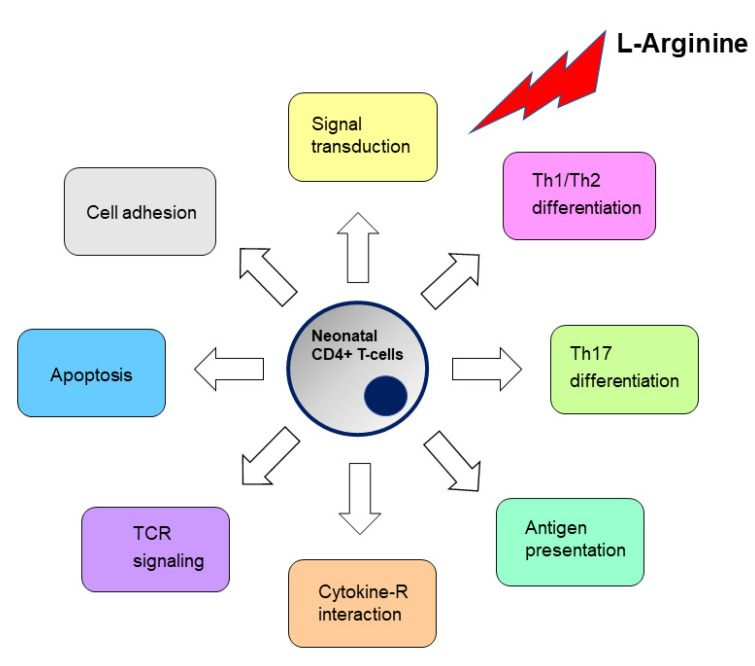
Regulatory effects of l-arginine on neonatal CD4^+^ T cells via DNA methylation modification.

Functional T cell regulation is crucial for maintaining homeostasis, and epigenetic modifications play an important role in development, polarization, and activation of lymphocytes. Examining genome-wide DNA-methylation patterns is a useful method for assessing specific gene regulation in particular cell types [36]. In the current study, we identified relative hypermethylated states in gene promoters of neonatal CD4^+^ T cells, compared to those of adults, that suggest more genes were inactive in neonatal CD4+ cells than in adult CD4^+^ cells. Down-regulated gene expression and protein production of IFN-γ and IL-17 corresponded to hypermethylation of their respective promoters in neonatal T cells. IL-4, however, demonstrated a seemingly contradictory pattern with relatively hypermethylated promoter regions but higher gene expression and protein production in neonatal CD4^+^ T cells, compared to those of adults. Regulatory effects of l-arginine on DNA methylation are gene-specific. Through DNA methylation modification, l-arginine supplementation exerts substantial effects on neonatal CD4^+^ T cell functions, including Th cell polarization, cell adhesion, apoptosis, T cell receptor signaling, cytokine production, cytokine receptor interaction, antigen presentation, and several signal transduction pathways (Figure 7).

Cord blood T cells showed a higher degree of DNA methylation of the IL-4 promoter but also higher IL-4 production. Similar phenomena were observed in other genes. Activated neonatal CD4^+^ T cells were shown to produce large-scale of changes in gene expression with limited changes in DNA methylation, suggesting that the inducible gene response is largely independent of changes in DNA methylation [37]. Previous study also showed that Th2 polarization was regulated both by demethylation and increased histone acetylation of the IL-4 gene which maintains an open chromatin conformation and increases the binding of polarizing transcription factors, such as STAT-6 and GATA-3 [38]. For example, the region of RHS4-7 in the naive T cells is hypoacetylated. Once stimulated by TCR, this region will preferentially be highly acetylated under Th2 polarization, which is consistent with its increased hypersensitivity [38].

Among the genes activated by DNA hypomethylation due to the l-arginine treatment, we were particularly interested in IL-13. IL-13 is a TH2-type cytokine that is predominantly produced by activated CD4+ Th2 T cell clones [39]. There is a close link between IL-4 and IL-13 activity: both activate the α-subunit of the IL-4 receptor and induce immunoglobulin E synthesis and eosinophils recruitment [39,40]. More and more evidence demonstrates in utero priming of cells of the fetal immune system to antigen [41,42]. Williams et al. have showed that IL-13 production is increased in 80% of fetal mononuclear cells with PHA stimulation [43]. It compatible with the observation that prenatal environment is important in modulating the immune response to antigen [44]. Modulatory effects of exogenous l-arginine supplementation can be validated by decreasing IL-13 gene promoter methylation and enhanced IL-13 mRNA and protein expression. IL-13 is involved in pathological mechanisms of several atopic and non-atopic processes [45,46]. As an effector, IL-13 helps counteract intracellular organisms including *Leishmania* spp. [47] and modulates tumor cell growth. IL-13 was also shown to regulate tissue repair and fibrosis through the extracellular matrix. We found that in human neonatal CD4^+^ T cells, the production of IL-13 is regulated by l-arginine supplementation through DNA methylation modification. Webster et al. showed that in neonatal CD4^+^ T cells, differential IL-13 expression depends on presence of a permissive chromatin structure at the proximal promoter in Th2 cells rather than on the formation of repressive chromatin in Th1 cells [48]. Our results are in line with this observation. Exogenous l-arginine supplementation may reduce the DNA methylation signature of the IL-13 gene promoter, enhance IL-13 gene expression, and elicit higher IL-13 production. Embryonic development in utero is essential for determining future susceptibility to disease, and the potential role of epigenetic processes in intrauterine programming of immunity has attracted a considerable interest. In general, DNA methylation leads to transcriptional repression by altering the recognition of transcription factors and by reducing the accessibility of DNA for transcription factors [49]. During embryonal development, DNA methylation is tightly regulated and modified. For normal embryo development and differentiation, DNA methylation is mostly eliminated after fertilization and reoccurs during early fetal development [50]. Cord blood leukocyte DNA methylation was suggested to be an observation window for fetal development [51]. Differentiation and polarization of CD4^+^ T cell subsets such as Th1, Th2, Th17 cells, and Treg are regulated by DNA methylation. For example, cytotoxic T lymphocyte–associated protein 4 (CTLA-4), CD25, and Treg-associated molecules are regulated by DNA demethylation [52]. In neonates, DNA methylation patterns appear to increase Th2 immune responses. A previous report has shown that cord blood CD4^+^ T cells are hypermethylated at CpG and non-CpG sites within and adjacent to the IFN-γ promoter, which corresponds to lower IFN-γ production in neonates compared to adults [29,30]; however, the CpG methylation status of IFN-γ promoter in cord blood CD8^+^ T cells and cord blood natural killer cells is comparable to that in adults [30]. DNA methylation plays an important role in maintaining low IFN-γ production in neonatal CD4^+^ T cells; In contrast, IL-4 production appears not to follow this pattern.

Thus, other mechanisms are also involved in Th2 polarization in neonates. GO analysis of cord blood mononuclear cells showed that methylation changes in genes were mainly associated with cell surface receptors and signal transduction [53]. Our study provides novel insights as we focused not only on CD4^+^ T cell maturation but also on functional changes due to mitogen stimulation.

Nutrition intervention is a promising strategy to modulate immune development and functioning. Nutrition epigenomics is a new research topic investigating the effects of diet on gene expression. Some nutritional factors such as folate, vitamins, and l-arginine can change DNA methylation [31,54]. In mice, DNA methylation of the promoters of CD4^+^ T cell-related genes were found to contribute to poor immune responses of neonates during pneumonia. The manifestation of T cell markers in neonatal mouse lungs was altered using a DNA hypomethylating agent [55]. In humans, hydrolyzed casein formula containing probiotics for infants was shown to elicit higher IL-4/IL-5 and a lower IL-10/IFN-γ DNA methylation patterns in CD4^+^ T cells, in contrast to soy formula [56]. Even prenatal probiotic treatments may change global DNA methylation patterns, and CD4^+^ T cells of newborn to tend to hypomethylation especially regarding PI3K/MAPK-, TGF-β-, and chemokine signaling-related genes [29]. In the current study, we determined the effects of exogenous l-arginine supplementation on DNA methylation pattern of neonatal Th cells at a genome-wide scale. We found that several genes were differentially methylated in relation to the development of Th1/Th2/Th17 polarization, encouraging us to build a classifier to assess the development of allergic disease.

## 5. Conclusions

In conclusion, the relative DNA hypermethylated status in neonatal T cells compared to that in adults can explain the naïve stage of neonatal immunity. DNA methylation partially accounts for the mechanism underlying differential immune function in neonates. Modulatory effects of l-arginine on DNA methylation are gene-specific. l-arginine supplementation decreased methylation in the IL-13 gene promoter region and enhanced IL-13 mRNA and protein production in neonatal Th cells. Nutritional intervention is a potential strategy to modulate immune function of neonates.

## Figures and Tables

**Table 1:** The summary of significantly altered CpG dinucleotides, genes and enriched pathways.

Comparison	CpG Dicuelcotides	Gene	Enriched Pathway
CT vs. CC	3797	1390	50
AT vs. AC	617	296	18
AC vs. CC	33,150	7730	24

## Data Availability

The data presented in this study are available on request from the corresponding author.

## Supplementary Materials

The following are available online at https://www.mdpi.com/article/10.3390/nu13082780/s1, Figure S1: Scatter plot of neonatal and adult CpG methylation. We determined β-values (methylation proportions) of CpG dinucleotides using M850K assays. Each CpG dinucleotide produced one βcc and one βac value. The former and the latter were derived from three CC and three AC samples, respectively, and both without l-arginine treatment. For each chromosome, we tabulated βcc and one βac values by sorting the βcc values. In total, the βcc and βac values of 865,859 CpG dinucleotides were highly correlated. (Pearson’s *r* = 0.9874; *p* < 0.0001). Figure S2. Comparisons of GO items between cord blood (CB) and adult blood (AD). We identified significantly hypomethylated CpG dinucleotides (CT vs. CC, comparing the factor l-arginine treatment) and identified activated genes of cord blood and adult blood samples, followed by GO analysis. We then compared the top-10 most significant GO items identified in either CB or AD samples. Table S1: The sequences of qPCR primers. 18S gene was applied as the internal control. Table S2: The result of pathway enrichment analysis on the genes with CpG markers significantly methylated between AC and CC samples. Table S3: The result of pathway enrichment analysis on the genes with CpG markers significantly methylated between AC and CC samples. Table S4: The result of pathway enrichment analysis on the genes with CpG markers significantly methylated between AT and AC samples. Table S5: The result of GO analysis on the genes with CpG markers significantly methylated between CT and CC samples. Table S6: The result of GO analysis on the genes with CpG markers significantly methylated between AT and AC samples.

## References

[1] Yu H.R., Huang Y.C., Yang K.D. (2003). Neonatal varicella frequently associated with visceral complications: A retrospective analysis. Acta Paediatr. Taiwanica Taiwan Er Ke Yi Xue Hui Za Zhi.

[2] Yang K.D., Hill H.R. (1996). Immune responses to infectious diseases: An evolutionary perspective. Pediatr. Infect. Dis. J..

[3] Yu H.R., Chang J.C., Chen R.F., Chuang H., Hong K.C., Wang L., Yang K.D. (2003). Different antigens trigger different Th1/Th2 reactions in neonatal mononuclear cells (MNCs) relating to T-bet/GATA-3 expression. J. Leukoc. Biol..

[4] Goldmann D.A. (1989). Prevention and management of neonatal infections. Infect. Dis. Clin. N. Am..

[5] Yu H.R., Kuo H.C., Huang H.C., Kuo H.C., Chen T.Y., Huang L.T., Tain Y.L., Chen C.C., Sheen J.M., Lin I.C. (2011). Identification of immunodeficient molecules in neonatal mononuclear cells by proteomic differential displays. Proteomics.

[6] Wu G., Bazer F.W., Davis T.A., Kim S.W., Li P., Marc Rhoads J., Carey Satterfield M., Smith S.B., Spencer T.E., Yin Y. (2009). Arginine metabolism and nutrition in growth, health and disease. Amino Acids.

[7] Wu G., Morris S.M. (1998). Arginine metabolism: Nitric oxide and beyond. Biochem. J..

[8] Wu G., Bazer F.W., Satterfield M.C., Li X., Wang X., Johnson G.A., Burghardt R.C., Dai Z., Wang J., Wu Z. (2013). Impacts of arginine nutrition on embryonic and fetal development in mammals. Amino Acids.

[9] Che D., Adams S., Zhao B., Qin G., Jiang H. (2019). Effects of Dietary l-arginine Supplementation from Conception to Post- Weaning in Piglets. Curr. Protein Pept. Sci..

[10] McKnight J.R., Satterfield M.C., Jobgen W.S., Smith S.B., Spencer T.E., Meininger C.J., McNeal C.J., Wu G. (2010). Beneficial effects of l-arginine on reducing obesity: Potential mechanisms and important implications for human health. Amino Acids.

[11] Yu H.R., Kuo H.C., Huang L.T., Chen C.C., Tain Y.L., Sheen J.M., Tiao M.M., Huang H.C., Yang K.D., Ou C.Y. (2014). l-Arginine modulates neonatal lymphocyte proliferation through an interleukin-2 independent pathway. Immunology.

[12] Batshaw M.L., Wachtel R.C., Thomas G.H., Starrett A., Brusilow S.W. (1984). Arginine-responsive asymptomatic hyperammonemia in the premature infant. J. Pediatr..

[13] Becker R.M., Wu G., Galanko J.A., Chen W., Maynor A.R., Bose C.L., Rhoads J.M. (2000). Reduced serum amino acid concentrations in infants with necrotizing enterocolitis. J. Pediatr..

[14] Zamora S.A., Amin H.J., McMillan D.D., Fick G.H., Butzner J.D., Parsons H.G., Scott R.B. (1998). Plasma l-arginine concentration, oxygenation index, and systemic blood pressure in premature infants. Crit. Care Med..

[15] Tomlinson C., Rafii M., Sgro M., Ball R.O., Pencharz P. (2011). Arginine is synthesized from proline, not glutamate, in enterally fed human preterm neonates. Pediatr. Res..

[16] Paul W.E., Seder R.A. (1994). Lymphocyte responses and cytokines. Cell.

[17] Yu H.R., Kuo H.C., Chen C.C., Sheen J.M., Tiao M.M., Chen Y.C., Chang K.A., Tain Y.L., Huang L.T. (2014). Prenatal dexamethasone exposure in rats results in long-term epigenetic histone modifications and tumour necrosis factor-alpha production decrease. Immunology.

[18] Yu H.R., Tain Y.L., Sheen J.M., Tiao M.M., Chen C.C., Kuo H.C., Hung P.L., Hsieh K.S., Huang L.T. (2016). Prenatal Dexamethasone and Postnatal High-Fat Diet Decrease Interferon Gamma Production through an Age-Dependent Histone Modification in Male Sprague-Dawley Rats. Int. J. Mol. Sci..

[19] Yu H.R., Huang L.H., Li S.C. (2018). Roles of microRNA in the immature immune system of neonates. Cancer Lett..

[20] Weitzel R.P., Lesniewski M.L., Haviernik P., Kadereit S., Leahy P., Greco N.J., Laughlin M.J. (2009). microRNA 184 regulates expression of NFAT1 in umbilical cord blood CD4^+^ T cells. Blood.

[21] Lederhuber H., Baer K., Altiok I., Sadeghi K., Herkner K.R., Kasper D.C. (2011). MicroRNA-146: Tiny player in neonatal innate immunity?. Neonatology.

[22] Charrier E., Cordeiro P., Cordeau M., Dardari R., Michaud A., Harnois M., Merindol N., Herblot S., Duval M. (2012). Post-transcriptional down-regulation of Toll-like receptor signaling pathway in umbilical cord blood plasmacytoid dendritic cells. Cell Immunol..

[23] Huang H.C., Yu H.R., Huang L.T., Huang H.C., Chen R.F., Lin I.C., Ou C.Y., Hsu T.Y., Yang K.D. (2012). miRNA-125b regulates TNF-alpha production in CD14^+^ neonatal monocytes via post-transcriptional regulation. J. Leukoc. Biol..

[24] Huang H.C., Yu H.R., Hsu T.Y., Chen I.L., Huang H.C., Chang J.C., Yang K.D. (2017). MicroRNA-142-3p and let-7g Negatively Regulates Augmented IL-6 Production in Neonatal Polymorphonuclear Leukocytes. Int. J. Biol. Sci..

[25] Yu H.R., Hsu T.Y., Huang H.C., Kuo H.C., Li S.C., Yang K.D., Hsieh K.S. (2016). Comparison of the Functional microRNA Expression in Immune Cell Subsets of Neonates and Adults. Front. Immunol..

[26] Brand S., Kesper D.A., Teich R., Kilic-Niebergall E., Pinkenburg O., Bothur E., Lohoff M., Garn H., Pfefferle P.I., Renz H. (2012). DNA methylation of TH1/TH2 cytokine genes affects sensitization and progress of experimental asthma. J. Allergy Clin. Immunol..

[27] Liu J., Lluis A., Illi S., Layland L., Olek S., von Mutius E., Schaub B. (2010). T regulatory cells in cord blood—FOXP3 demethylation as reliable quantitative marker. PLoS ONE.

[28] White G.P., Hollams E.M., Yerkovich S.T., Bosco A., Holt B.J., Bassami M.R., Kusel M., Sly P.D., Holt P.G. (2006). CpG methylation patterns in the IFNgamma promoter in naive T cells: Variations during Th1 and Th2 differentiation and between atopics and non-atopics. Pediatr. Allergy Immunol..

[29] Forsberg A., Huoman J., Soderholm S., Bhai Mehta R., Nilsson L., Abrahamsson T.R., Ernerudh J., Gustafsson M., Jenmalm M.C. (2020). Pre- and postnatal Lactobacillus reuteri treatment alters DNA methylation of infant T helper cells. Pediatr. Allergy Immunol..

[30] White G.P., Watt P.M., Holt B.J., Holt P.G. (2002). Differential patterns of methylation of the IFN-gamma promoter at CpG and non-CpG sites underlie differences in IFN-gamma gene expression between human neonatal and adult CD45RO^−^ T cells. J. Immunol..

[31] Yu H.R., Tsai C.C., Chang L.S., Huang H.C., Cheng H.H., Wang J.Y., Sheen J.M., Kuo H.C., Hsieh K.S., Huang Y.H. (2017). L-Arginine-Dependent Epigenetic Regulation of Interleukin-10, but Not Transforming Growth Factor-beta, Production by Neonatal Regulatory T Lymphocytes. Front. Immunol..

[32] Huang L.H., Kuo H.C., Pan C.T., Lin Y.S., Huang Y.H., Li S.C. (2018). Multiomics analyses identified epigenetic modulation of the S100A gene family in Kawasaki disease and their significant involvement in neutrophil transendothelial migration. Clin. Epigenet..

[33] Uebelhoer L.S., Lancioni C.L. (2018). CD4^+^ T Cell Activation During the Newborn Period: Barriers Against and Pathways toward Th1 Immunity. Crit. Rev. Immunol..

[34] Tsafaras G.P., Ntontsi P., Xanthou G. (2020). Advantages and Limitations of the Neonatal Immune System. Front. Pediatr..

[35] Rodriguez P.C., Zea A.H., Culotta K.S., Zabaleta J., Ochoa J.B., Ochoa A.C. (2002). Regulation of T cell receptor CD3zeta chain expression by l-arginine. J. Biol. Chem..

[36] Schmidl C., Delacher M., Huehn J., Feuerer M. (2018). Epigenetic mechanisms regulating T-cell responses. J. Allergy Clin. Immunol..

[37] Martino D., Maksimovic J., Joo J.H., Prescott S.L., Saffery R. (2012). Genome-scale profiling reveals a subset of genes regulated by DNA methylation that program somatic T-cell phenotypes in humans. Genes Immun..

[38] Lee G.R., Kim S.T., Spilianakis C.G., Fields P.E., Flavell R.A. (2006). T helper cell differentiation: Regulation by cis elements and epigenetics. Immunity.

[39] Bagnasco D., Ferrando M., Varricchi G., Passalacqua G., Canonica G.W. (2016). A Critical Evaluation of Anti-IL-13 and Anti-IL-4 Strategies in Severe Asthma. Int. Arch. Allergy Immunol..

[40] Junttila I.S. (2018). Tuning the Cytokine Responses: An Update on Interleukin (IL)-4 and IL-13 Receptor Complexes. Front. Immunol..

[41] Piccinni M.P., Mecacci F., Sampognaro S., Manetti R., Parronchi P., Maggi E., Romagnani S. (1993). Aeroallergen sensitization can occur during fetal life. Int. Arch. Allergy Immunol..

[42] Chung E.K., Miller R.L., Wilson M.T., McGeady S.J., Culhane J.F. (2007). Antenatal risk factors, cytokines and the development of atopic disease in early childhood. Arch. Dis. Child Fetal Neonatal Ed..

[43] Loibichler C., Pichler J., Gerstmayr M., Bohle B., Kisst H., Urbanek R., Szepfalusi Z. (2002). Materno-fetal passage of nutritive and inhalant allergens across placentas of term and pre-term deliveries perfused in vitro. Clin. Exp. Allergy.

[44] Williams T.J., Jones C.A., Miles E.A., Warner J.O., Warner J.A. (2000). Fetal and neonatal IL-13 production during pregnancy and at birth and subsequent development of atopic symptoms. J. Allergy Clin. Immunol..

[45] MacGillivray D.M., Kollmann T.R. (2014). The role of environmental factors in modulating immune responses in early life. Front. Immunol..

[46] Wills-Karp M., Luyimbazi J., Xu X., Schofield B., Neben T.Y., Karp C.L., Donaldson D.D. (1998). Interleukin-13: Central mediator of allergic asthma. Science.

[47] Marone G., Granata F., Pucino V., Pecoraro A., Heffler E., Loffredo S., Scadding G.W., Varricchi G. (2019). The Intriguing Role of Interleukin 13 in the Pathophysiology of Asthma. Front. Pharmacol..

[48] Zheng T., Zhu Z., Wang Z., Homer R.J., Ma B., Riese R.J., Chapman H.A., Shapiro S.D., Elias J.A. (2000). Inducible targeting of IL-13 to the adult lung causes matrix metalloproteinase- and cathepsin-dependent emphysema. J. Clin. Investig..

[49] Wynn T.A. (2003). IL-13 effector functions. Annu. Rev. Immunol..

[50] Webster R.B., Rodriguez Y., Klimecki W.T., Vercelli D. (2007). The human IL-13 locus in neonatal CD4^+^ T cells is refractory to the acquisition of a repressive chromatin architecture. J. Biol. Chem..

[51] Jaenisch R., Bird A. (2003). Epigenetic regulation of gene expression: How the genome integrates intrinsic and environmental signals. Nat. Genet..

[52] Reynolds R.M., Jacobsen G.H., Drake A.J. (2013). What is the evidence in humans that DNA methylation changes link events in utero and later life disease?. Clin. Endocrinol..

[53] Dzierzak E., Speck N.A. (2008). Of lineage and legacy: The development of mammalian hematopoietic stem cells. Nat. Immunol..

[54] Ohkura N., Hamaguchi M., Morikawa H., Sugimura K., Tanaka A., Ito Y., Osaki M., Tanaka Y., Yamashita R., Nakano N. (2012). T cell receptor stimulation-induced epigenetic changes and Foxp3 expression are independent and complementary events required for Treg cell development. Immunity.

[55] Martino D.J., Tulic M.K., Gordon L., Hodder M., Richman T.R., Metcalfe J., Prescott S.L., Saffery R. (2011). Evidence for age-related and individual-specific changes in DNA methylation profile of mononuclear cells during early immune development in humans. Epigenetics.

[56] Canani R.B., Costanzo M.D., Leone L., Bedogni G., Brambilla P., Cianfarani S., Nobili V., Pietrobelli A., Agostoni C. (2011). Epigenetic mechanisms elicited by nutrition in early life. Nutr. Res. Rev..

