# Supplementary material for: The Functional DNA Methylation Signatures Relevant to Altered Immune Response of Neonatal T Cells with l-Arginine Supplementation
Source: Nutrients. 2021 Aug 13;13(8):2780. doi: 10.3390/nu13082780 (PMC8401784; doi:10.3390/nu13082780)
Supplement: Supplementary file 1 [file nutrients-13-02780-s001.zip › Supplementary Table 1.pdf]

Supplementary Table S1. The sequences of qPCR primers. 18S gene was applied as the internal control.

Gene symbol	Forward primer	Reverse primer
IFN-γ	5'- CTA ATT ATT CGG TAA CTG ACT TGA -3'	5'-ACA GTT CAG CCA TCA CTT GGA-3'
IL-4	5'-ACT TTG AAC AGC CTC ACA GAG-3'	5'-TTG GAG GCA GCA AAG ATG TC-3'
IL-13	5'- TGA GGA GCT GGT CAA CAT CA 3'	5'- CAG GTT GAT GCT CCA TAC CAT-3'
IL-17A	5'- CAT CCA TAA CCG GAA TAC CAA TA-3'	5'- TAG TCC ACG TTC CCA TCA GC-3'
18S	5'-CGCAGCTAGGAATAATGGAATAGG-3'	5'-CATGGCCTCAGTTCCGAAA-3'
