# Supplementary figures and images for: The Functional DNA Methylation Signatures Relevant to Altered Immune Response of Neonatal T Cells with l-Arginine Supplementation
Source: Nutrients. 2021 Aug 13;13(8):2780. doi: 10.3390/nu13082780 (PMC8401784; doi:10.3390/nu13082780)

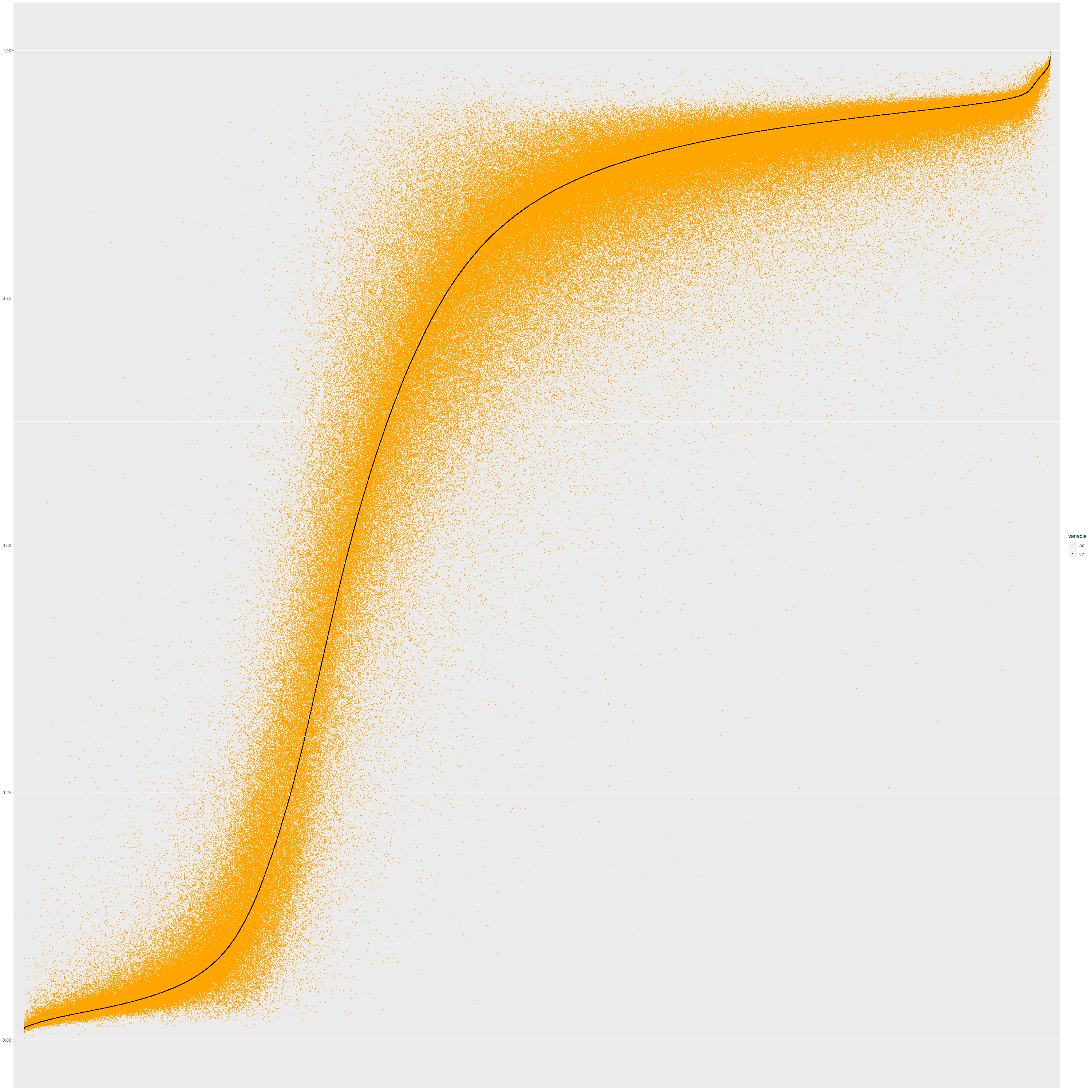

Supplement: Supplementary file 1 [file nutrients-13-02780-s001.zip › Supplementary figure 1.tiff]
